# Rectal sensitivity correlated with gastrointestinal‐mediated glucose disposal, but not the incretin effect

**DOI:** 10.1002/edm2.463

**Published:** 2023-12-07

**Authors:** Sondre Meling, Erling Tjora, Heike Eichele, Rasmus B. Nedergaard, Filip K. Knop, Niels Ejskjaer, Siri Carlsen, Pål R. Njølstad, Christina Brock, Eirik Søfteland

**Affiliations:** ^1^ Department of Medicine Stavanger University Hospital Stavanger Norway; ^2^ Department of Clinical Science University of Bergen Bergen Norway; ^3^ Children and Youth Clinic Haukeland University Hospital Bergen Norway; ^4^ Department of Biological and Medical Psychology, Faculty of Psychology University of Bergen Bergen Norway; ^5^ Regional resource Centre for Autism, ADHD and Tourette Syndrome Western Norway, Division of Psychiatry Haukeland University Hospital Bergen Norway; ^6^ Mech‐Sense, Department of Gastroenterology and Hepatology Aalborg University Hospital Aalborg Denmark; ^7^ Center for Clinical Metabolic Research Copenhagen University Hospital—Herlev and Gentofte Copenhagen Denmark; ^8^ Department of Clinical Medicine, Faculty of Health and Medical Sciences University of Copenhagen Copenhagen Denmark; ^9^ Steno Diabetes Center Copenhagen Gentofte Denmark; ^10^ Novo Nordisk Foundation Center for Basic Metabolic Research University of Copenhagen Copenhagen Denmark; ^11^ Department of Clinical Medicine, Faculty of Medicine Aalborg University Hospital Aalborg Denmark; ^12^ Steno Diabetes Center North Denmark Aalborg University Hospital Aalborg Denmark; ^13^ Department of Endocrinology Aalborg University Hospital Aalborg Denmark; ^14^ Mohn Center for Diabetes Precision Medicine, Department of Clinical Science University of Bergen Bergen Norway; ^15^ Department of Medicine Haukeland University Hospital Bergen Norway

**Keywords:** diabetic autonomic neuropathy, evoked potentials, gastrointestinal‐mediated glucose disposal, incretin effect, rectal balloon distention, rectal hyposensitivity, type 2 diabetes

## Abstract

**Objective:**

The mechanisms behind the diminished incretin effect in type 2 diabetes are uncertain, but impaired vagal transmission has been suggested. We aimed to investigate the association between the incretin effect and autonomic neuropathy, and the degree of dysglycaemia and duration of diabetes.

**Design and Methods:**

For a cross‐sectional study, we included participants with either longstanding type 2 diabetes, recent onset, untreated diabetes and controls without diabetes matched for age, sex and body mass index. Autonomic nerve function was assessed with cardiovascular reflex tests, heart rate variability and sudomotor function. Visceral afferent nerves in the gut were tested performing rapid rectal balloon distention. An oral glucose tolerance test and an intravenous isoglycaemic glucose infusion were performed to calculate the incretin effect and gastrointestinal‐mediated glucose disposal (GIGD).

**Results:**

Sixty‐five participants were recruited. Participants with diabetes had rectal hyposensitivity for earliest sensation (3.7 ± 1.1 kPa in longstanding, 4.0 ± 1.3 in early), compared to controls (3.0 ± 0.9 kPa), *p* = .005. Rectal hyposensitivity for earliest sensation was not associated with the incretin effect (rho = −0.204, *p* = .106), but an association was found with GIGD (rho −0.341, *p* = .005). Incretin effect and GIGD were correlated with all glucose values, HbA1c and duration of diabetes.

**Conclusions:**

Rectal hyposensitivity was uncovered in both longstanding and early type 2 diabetes, and was not associated with the incretin effect, but with GIGD, implying a potential link between visceral neuropathy and gastrointestinal handling of glucose. Both the incretin effect and GIGD were associated with the degree of dysglycaemia and the duration of diabetes.

**Previously Published:**

Some of the data have previously been published and presented as a poster on the American Diabetes Association 83rd Scientific Sessions: Meling et al; 1658‐P: Rectal Hyposensitivity, a Potential Marker of Enteric Autonomic Nerve Dysfunction, Is Significantly Associated with Gastrointestinally Mediated Glucose Disposal in Persons with Type 2 Diabetes. *Diabetes* 20 June 2023; 72 (Supplement_1): 1658–P. https://doi.org/10.2337/db23‐1658‐P.

## Graphical abstract

We demonstrate for the first time a visceral hyposensitivity of the gut in an early stage of diabetes, and find an association between visceral hyposensitivity and the gastrointestinal‐mediated glucose disposal, but not the incretin effect per se. The findings indicate that intact neuronal transmission is not a prerequisite for the incretin effect, but should lead to further studies on the potential role of the autonomic nervous system in other aspects involved in gastrointestinal‐mediated glucose disposal.

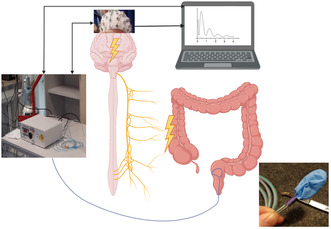

## 1 INTRODUCTION

The incretin effect refers to the amplified insulin secretion when glucose is ingested orally compared to administered intravenously.^1^ The two main incretin hormones involved, glucagon‐like peptide 1 (GLP‐1) and glucose‐dependent insulinotropic polypeptide (GIP) are secreted from enteroendocrine cells in response to food ingestion.

It is well established that the incretin effect plays an important role in normal glucose tolerance, and also that a diminished incretin effect is one of several pathomechanistic components of type 2 diabetes.^2^ Importantly, however, there is uncertainty as to whether the diminished incretin response in type 2 diabetes is a cause or a consequence of the condition.^3^ It is also unknown why the effect is diminished, with several studies having concluded that patients with type 2 diabetes have normal levels of total GLP‐1 responses during oral glucose or liquid meal tests.^4^, ^5^ Thus, other underlying mechanisms should explain the diminished incretin effect.

Bioactive GLP‐1 has a half‐life of less than 2 min, with ≥50% of secreted GLP‐1 being inactivated before entering systemic circulation. This rapid degradation has led to the hypothesis that GLP‐1 may act locally at vagal nerve terminals innervating the portal vein, before being degraded. There is also support for the activation of sensory vagal afferents expressing GLP‐1 receptors found in the gut, with transmission to the nucleus of the solitary tract in the brain stem, further reaching the hypothalamus and thus influencing central autonomic regulation.^6^ Different lesions at these sites of vagal transmission have been shown to abolish satiation, increase meal size, induce postprandial hyperglycaemia and increase gastric emptying rate.^7^, ^8^ Until recently, the effects of GLP‐1 in human physiology were the most extensively studied; however, recently GIP has been indicated as perhaps the most important for postprandial glycaemic control.^9^ Evidence also support that endogenous GIP has a progressive loss of insulinotropic and glucose‐lowering actions from health to obesity to type 2 diabetes.^10^

A deeper understanding of the underlying mechanisms of the diminished incretin effect could lead to new treatment targets, and provide an understanding that could help in treatment individualisation. With an increased awareness of autonomic neuropathy in the early stages of diabetes, even in prediabetes, the main aim of the current study was to investigate if autonomic neuropathy was associated with the incretin effect.^11^, ^12^ Secondary aims were to investigate associations between the incretin effect, degree of dysglycaemia and duration of diabetes.

## 2 MATERIALS AND METHODS

A cross‐sectional study was conducted including one group of people with type 2 diabetes for more than 10 years (*longstanding diabetes group*), one group with recently diagnosed (within 1 year), untreated type 2 diabetes (*early diabetes group*) and one group without diabetes (*control group*). Participants were recruited mainly through regional newspaper advertisements. Those with longstanding diabetes were classified based on self‐reported duration, and an oral glucose tolerance test (OGTT) confirmed the diagnosis. The two latter groups were recruited to match the longstanding diabetes group for age, sex and body mass index (BMI), and participants were further classified into early diabetes or controls according to the American Diabetes Association (ADA) criteria based on the OGTT.^13^ All investigations were performed at a single centre (Bergen, Norway). Exclusion criteria were major abdominal surgery, rectosigmoid disease interfering with sensitivity, chronic pancreatitis, uremic condition (eGFR<30 mL/min), atrial fibrillation or other major dysrhythmias, cardiac pacemaker, proliferative retinopathy or present use of a GLP‐1 receptor agonist or insulin.

Participants were instructed to avoid alcohol, sedatives or stimulant drugs 24 h before the nerve tests. Other medications were taken as normal. Food, coffee, tea and nicotine were avoided 3 h before the examination. The participants fasted, including medications and nicotine products, for a minimum of 10 h before the OGTT and intravenous isoglycaemic glucose infusion (IIGI) with antidiabetic medication withdrawn 3 days ahead.

### 2.1 Neuronal phenotyping

For cardiovascular reflex tests (CARTs) and heart rate variability (HRV) scores, we used the Vagus™ Device (Medicus Engineering, Aarhus, Denmark). Blood pressure was measured after 5 min rest, in response to standing, and after 1‐ and 3 min standing, using the WelchAllyn Connex ProBP 3400™ (EMEAI, Leiden, The Netherlands). Orthostatic hypotension was defined as a drop in systolic blood pressure >20 mmHg or diastolic blood pressure >10 mmHg. Stages of cardiovascular autonomic neuropathy (CAN) based on CARTs were defined as borderline if one ratio was abnormal and as definite or confirmed if two or three ratios were abnormal. CAN was defined as severe or advanced if the latter was combined with orthostatic hypotension.^11^

The Sudoscan™ device (Impeto Medical, Paris, France) was used to test the sudomotor function of hands and feet. The device reports absolute values for electrochemical skin conductance and categorical interpretations into normal function, moderately reduced or severely reduced function.

### 2.2 Oral glucose tolerance test

A cannula was placed in a cubital vein, with the forearm on the same side placed in a heating cuff to ensure arterialised blood. Fasting blood samples were taken at −30, −15 and 0 min. Within 5 min, the participants ingested a 250 mL solution of 75 g anhydrate glucose (Finnomedical, Finland) with 1.5 g of paracetamol (Karo Pharma Aktiebolag, Stockholm, Sweden). Blood samples (serum and EDTA blood) were then collected at 10, 20, 30, 50, 70, 90, 120, 150, 180 and 240 min. Glucose was measured simultaneously and at 5, 15, 25, 40, 60, 80, 105 and 135 min, using the HemoCue Glucose 201 DM RT (HemoCue, Angelholm, Sweden).

### 2.3 Intravenous isoglycaemic glucose infusion

Two cannulas were placed in cubital veins, one in each arm. Blood was sampled, and glucose was measured at the same intervals as the OGTT. At 0 min, a 200 mg/mL Glucose infusion (B. Braun, Melsungen AG, Germany) was initiated. The infusion rate was adjusted at each time point to duplicate the plasma glucose profile of the OGTT, and the total amount of administered glucose was noted. For glucose values in the OGTT and IIGI, we accepted a maximum of 20% difference, measured by area under the curve (AUC).

From the OGTT and IIGI, we calculated the following outcomes: The incretin effect, estimated from c‐peptide: [(AUC c peptideOGTT‐AUC c peptide IIGI)/AUC c‐peptide OGTT] × 100%, and the gastrointestinal‐mediated glucose disposal (GIGD): [(glucose OGTT‐glucose IIGI)/glucose = OGTT] × 100%. The paracetamol AUC of OGTT (μmol/L × min at 70 min) was calculated as a proxy for gastric emptying.^14^

### 2.4 Visceral sensitivity: Evoked brain potentials following rapid rectal balloon distention

**FIGURE 1:**
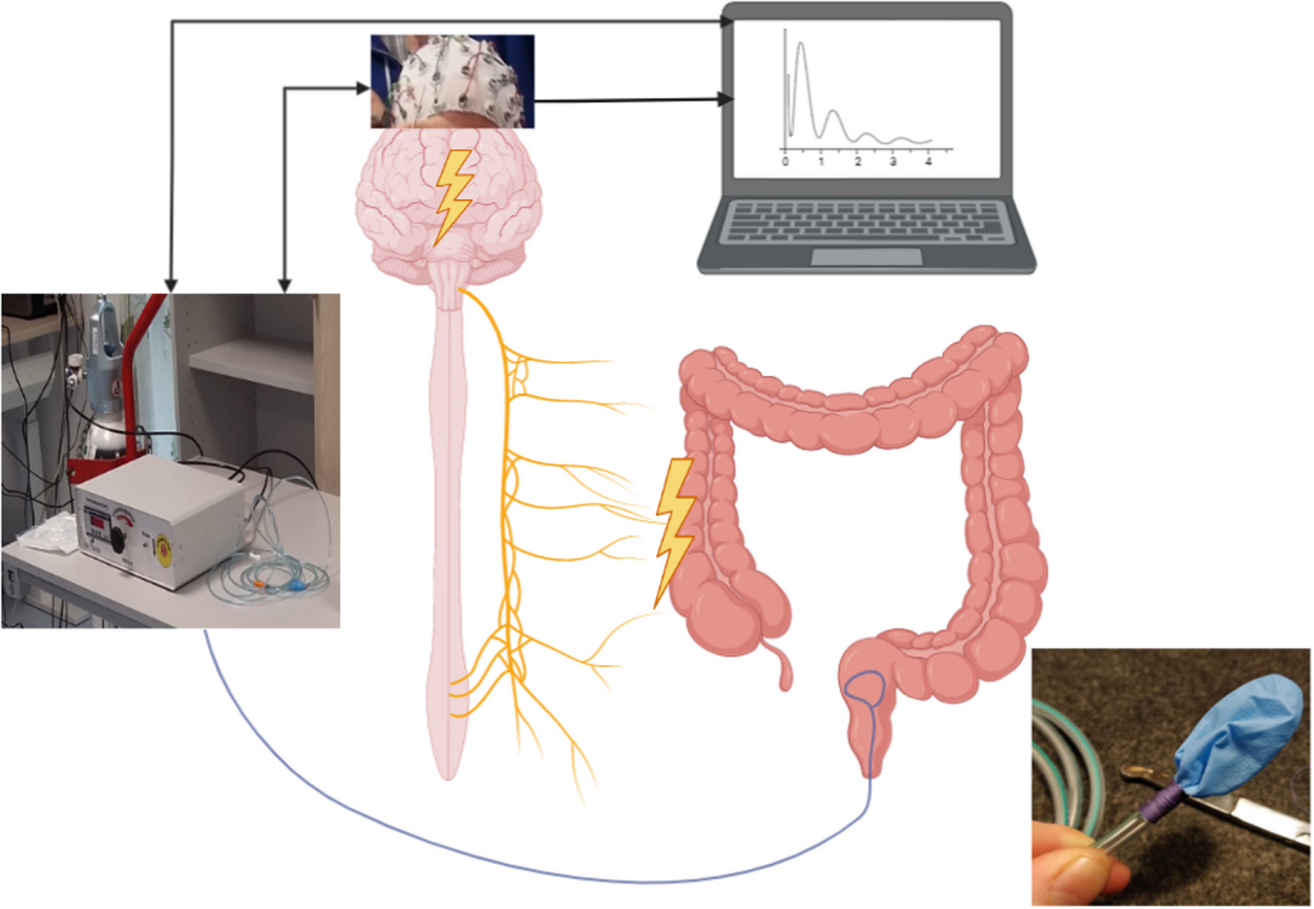
Setup for measuring evoked potentials following rapid rectal balloon distention. Figure created using Biorender.com, photographs by S. Meling.

The equipment and protocol are based on recent studies and have previously been described in more detail.^15^, ^16^, ^17^ Figure 1 shows the setup for the examination. A rectal balloon was placed 15 cm above the anal verge. A distinct and short stimulus was used; 150 ms inflation, followed by an instant deflation. A random interstimulus interval of 8 ± 2 s was enforced. Recorded outcomes were pressure needed to induce the earliest sensation and unpleasant threshold, and latency and amplitude of the evoked potentials.

### 2.5 Data analysis and statistics

In this explorative pilot project, a formal power analysis was unfeasible, mainly due to the unknown effect size for the rapid rectal balloon distention test. However, using effect sizes from previous studies on visceral sensitivity, the lowest number of participants needed to achieve a power of 0.8 would be 15 participants in each group.^18^, ^19^ Normally distributed data are provided as means with SD and skewed data as median with IQR. If equal variance was present, we used one‐way ANOVA, otherwise the Welch test for unequal variance when testing for statistical significance. For post hoc testing, we used the Bonferroni test where equal variance, and the Games–Howell test for unequal variance. Kruskal–Wallis test was used for statistical significance in the case of skewed data. For categorical data, the *p*‐value was calculated using the chi‐square test. For correlation analyses, we used Spearman's rank‐order correlation test. The AUCs were calculated using the trapezoidal rule. Missing values for c‐peptide and paracetamol were imputed from the average values before and after the missing values. For other missing data, subjects were removed from that analysis. Statistical significance was defined as a *p*‐value ≤.05, and SPSS Version: 28.0.1.0. (IBM, New York, US) was used.

## 3 RESULTS

### 3.1 Subjects

We recruited a total of 65 participants with a mean age of 69 years, 52% women, all Caucasians. For clinical characteristics, see Table 1.

**TABLE 1:** Clinical characteristics at baseline.

Clinical characteristics	Longstanding diabetes, *n* = 20	Early diabetes, *n* = 15	Controls, *n* = 30	*p*‐Value
Age (years at recruitment)	68.9 ± 7.8	69.3 ± 5.5	69.5 ± 6.2	.950
Sex (women/men), *n*	9/11	8/7	16/14	.911
BMI (kg/m^2^)	26.5 ± 4.4	25.7 ± 4.1	25.5 ± 3.8	.680
Diabetes duration (years)	16.8 ± 4.9	0	0	n/a
Total cholesterol, mmol/L	4.2 ± 0.8^a^	4.5 ± 1.2^a^	5.5 ± 1.0	<.001
HDL, mmol/L	1.3 ± 0.3^a^	1.4 ± 0.4^a^	1.9 ± 0.5	<.001
LDL, mmol/L	2.4 ± 0.6^a^	2.8 ± 1.1	3.3 ± 0.8	.001
Triglycerides, mmol/L	1.7 ± 1.3^a^	1.3 ± 0.5	1.0 ± 0.4	.009
eGFR, mL/min per 1.73 m^2^	84.9 ± 13.5	82.3 ± 11.7	80.3 ± 12.3	.458
Systolic blood pressure rest, mmHg	135 ± 15^b^	152 ± 14	139 ± 20^b^	.015
Diastolic blood pressure, rest, mmHg	80 ± 6^b^	86 ± 7	81 ± 7	.023
*Comorbidity*				
Nephropathy	0	0	0	n/a
Distal neuropathy, %	4.8	6.7	0	.400
Hypertension, %	52	47	17	.017
Cardiovascular disease, %	4.8	13	3.3	.401
*Drugs*				
Metformin, %	81	0	0	n/a
Sulphonylurea, %	19	0	0	n/a
DPP‐4 inhibitor, %	48	0	0	n/a
SGLT2 inhibitor, %	38	0	0	n/a
Other antidiabetic medication, %	9.5	0	0	n/a
Diet‐treated diabetes, %	9.5	100	0	n/a
Betablocker, %	4.8	20	13	.370
ACE‐inhibitor/ARB, %	48	40	10	<.001
Other antihypertensive medication, %	19	13	7	.410
Lipid modifying treatment, %	67	47	13	<.001
Smoking status, % (present/past/never)	10/38/52	7/13/80	3/43/54	.300

*Note*: Data are presented as *n*, % or mean ± standard deviation. Diabetes duration, comorbidity, smoking status and drugs are self‐reported. 80% of early diabetes was newly discovered, and diabetes duration was therefore set to zero. All other significant for all groups.

Abbreviations: ARB, angiotensin II receptor blocker; BMI, body mass index; DPP‐4, dipeptidyl peptidase‐4; eGFR, estimated glomerular filtration rate; HDL, high‐density lipoprotein; LDL, low‐density lipoprotein; SGLT‐2, sodium‐glucose co‐transporter 2.

^a^ Significant compared to controls.

^b^ Significant compared to early diabetes.

### 3.2 Between‐group differences

Neuronal phenotyping results have been previously published, but are summarily presented, as they are needed for further analyses in the present paper.^17^

Participants with diabetes showed rectal hyposensitivity, evident as higher pressure needed to elicit earliest sensation: Longstanding (3.7 ± 1.1 kPa); early (4.0 ± 1.3 kPa) compared to controls (3.0 ± 0.9 kPa), *p* = .005. There was no difference in pressure required to reach an unpleasant threshold, nor in evoked potential latencies or amplitudes.

Neuronal phenotyping revealed two cases of definite CAN. Proportions of definite or borderline CAN were as follows: longstanding diabetes 31%, early diabetes 23% and 17% of controls, *p* =0.537. No between‐group differences were found for HRV or sudomotor function.

**FIGURE 2:**
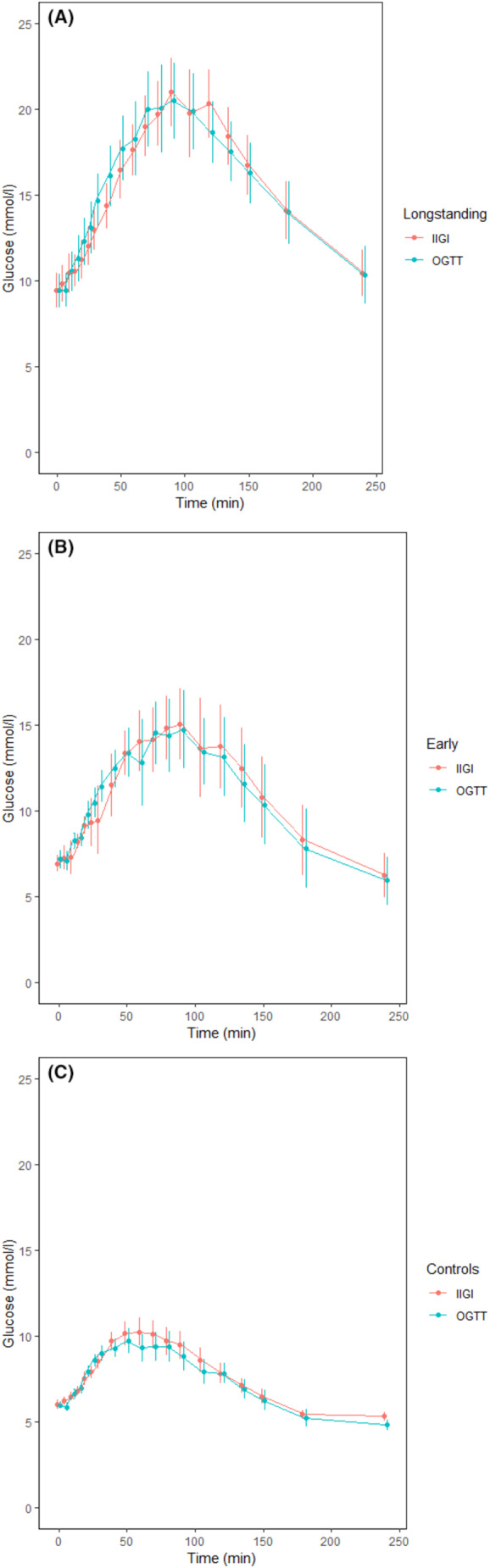
The difference between glucose values performing the OGTT and IIGI. (A) Longstanding diabetes, AUC: OGTT 3742 ± 834 mmol/L, IIGI 3730 ± 705 mmol/L, difference 5.8 ± 5.6%. (B) Early diabetes, AUC: OGTT 2521 ± 668 mmol/L, IIGI 2565 ± 694 mmol/L, difference 5.6 ± 3.5%. (C) Controls, AUC: OGTT 1707 ± 223 mmol/L, IIGI 1776 ± 186 mmol/L, difference 6.6 ± 4.3%.

The fasting and 2‐h glucose levels following the OGGT, and the HbA1c differed significantly between groups (Table 2). The mean differences between glucose in the OGTT and IIGI are shown in Figure 2A–C.

**TABLE 2:** Between‐group differences.

	Longstanding diabetes	Early diabetes	Controls	*p‐*Value
*Glucose, OGTT*	*n* = 20	*n* = 15	*n* = 30	
Baseline, mmol/L	9.4 ± 2.1	7.2 ± 1.0	6.0 ± 0.6	<.001
2‐h, mmol/L	18.7 ± 3.9	13.1 ± 4.2	7.9 ± 1.5	<.001
HbA1c, mmol/mol	53.5 ± 11.2	43.3 ± 4.9	37.1 ± 3.0	<.001
HbA1c, %	7.1 ± 1.0	6.1 ± 0.4	5.5 ± 0.3	<.001
*C‐peptide, nmol/L*	*n* = 18	*n* = 15	*n* = 30	
AUC, 240 min, OGTT	510 ± 271^a^	804 ± 280	664 ± 262	.009
AUC, 240 min, IIGI	438 ± 258	562 ± 238^b^	339 ± 170	.007
Incretin effect, %	12 ± 22^b^	30 ± 20^b^	48 ± 17	<.001
GIGD, %	17 ± 22	36 ± 15	59 ± 14	<.001
*Rapid rectal balloon distention*	*n* = 20	*n* = 15	*n* = 30	
Pressure first sensation, kPa (mmHg)	3.7 ± 1.1^b^ (28)	4.0 ± 1.3^b^ (30)	3.0 ± 0.9 (23)	.005
*Sudomotor function*				
Hands, μSiemens	65.4 ± 14.7	67.2 ± 14.1	71.3 ± 15.2	.367
Normal/moderate reduced/severely reduced, %	68/26/5	67/33/0	77/20/3	.797
Feet, μSiemens	73.0 ± 12.4	77.7 ± 6.7	75.3 ± 13.5	.508
Normal/moderate reduced/severely reduced, %	70/20/10	80/20/0	87/7/7	.419
Orthostatic hypotension, %	14.3	20.0	16.7	.902
CARTs				
	*n* = 19	*n* = 14	*n* = 28	
RS ratio	1.07 (1.02–1.11)	1.09 (1.04–1.15)	1.08 (1.06–1.15)	.266
Abnormal RS ratio, %	6.3	6.7	6.7	.960
	*n* = 19	*n* = 14	*n* = 26	
EI ratio	1.17 (1.05–1.31)	1.14 (1.07–1.23)	1.13 (1.11–1.23)	.787
Abnormal EI ratio, %	25	20	6.7	.340
	*n* = 16	*n* = 13	*n* = 23	
VM ratio	1.43 (1.23–1.67)	1.41 (1.32–1.48)	1.47 (1.33–1.62)	.314
Abnormal VM ratio	0	6.7	3.3	.550
CAN	*n* = 16	*n* = 13	*n* = 23	
No/borderline/definite, %	67/33/0	77/15/8	83/13/4	.487

*Note*: Data are provided as percentage, mean ± standard deviation or median with (interquartile range). Two participants were excluded from the analysis of the incretin effect, one due to an OGGT/IIGI glucose AUC difference above 20%, and one due to errors in c‐peptide measurements, both from the longstanding diabetes group. The reduction in the number of participants performing CARTs where mainly due to participants being unable to perform the Valsalva manoeuvre. RS ratio, the ratio between maximum HR within the first 15 s after standing up and minimum HR within the first 30 s after standing up; EI‐ratio, the mean ratio between the longest and shortest RR‐interval during deep respiration; VM ratio, the ratio between maximum heart rate at the end of forced respiration and minimal heart rate during inspiration‐expiration in rest appr. 30 s after releasing pressure.

Abbreviations: AUC, area under the curve; CAN, cardiovascular autonomic neuropathy; CARTs, cardiovascular autonomic reflex tests; GIGD, gastrointestinal‐mediated glucose disposal; HbA1c, haemoglobin A1c; IIGI, intravenous isoglycaemic glucose infusion; OGTT, oral glucose tolerance test.

^a^ Significant compared to early diabetes.

^b^ Significant compared to controls. All other significant between all groups (range <0.001–0.028).

**FIGURE 3:**
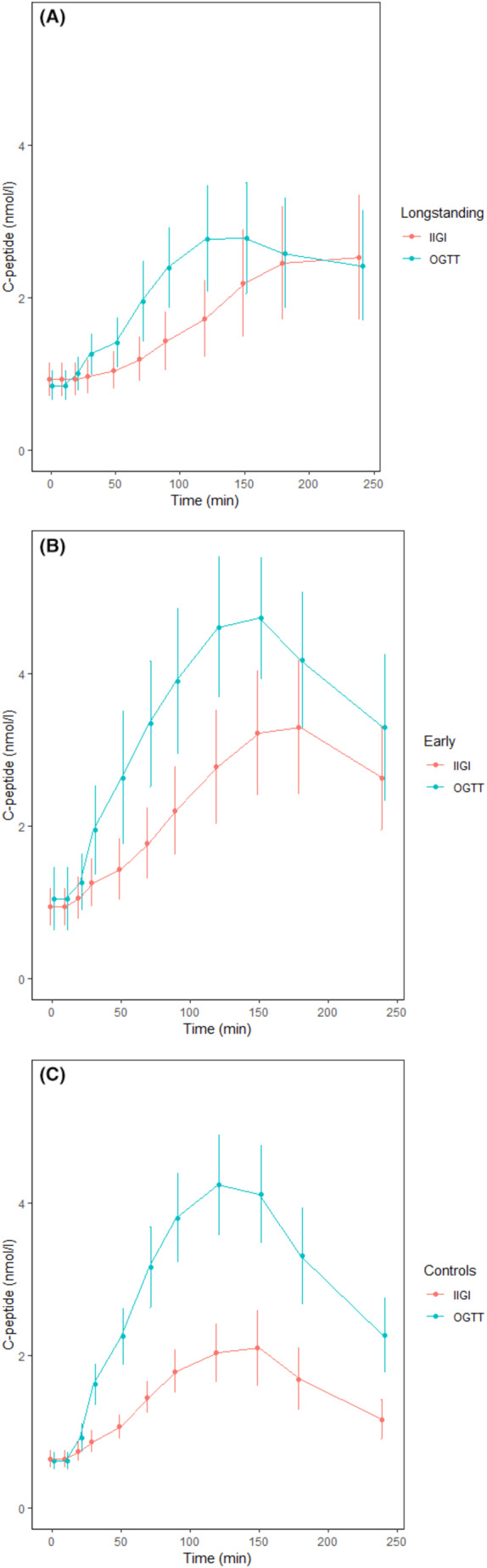
The incretin effect. (A) Longstanding diabetes, c‐peptide AUC values are found in Table 2. (B) Early diabetes, c‐peptide AUC values are found in Table 2. (C) Controls, c‐peptide AUC values are found in Table 2.

The incretin effect was significantly different for both diabetes groups (longstanding 12% ± 22%, early 30% ± 20%) compared to controls (48% ± 17%), *p* < .001, while GIGD was significantly different between all groups (longstanding diabetes 17% ± 22%, early diabetes 36% ± 15% and healthy controls 59% ± 14%), *p* < .001 (Table 2 and Figure 3A–C).

**FIGURE 4:**
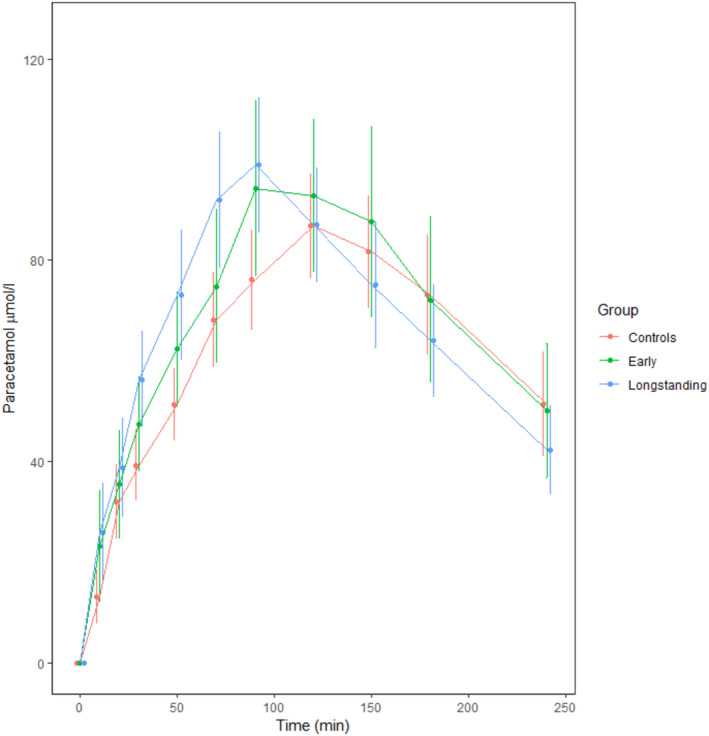
Paracetamol absorption test for the different groups. AUC at 70 min, μmol/L: Longstanding diabetes; 3868 ± 1320, early diabetes; 3256 ± 1151, controls; 2695 ± 1100, *p* = .004. Significant for longstanding diabetes compared to controls, *p* = .003.

Paracetamol AUC concentration was significantly higher for those with longstanding diabetes compared to controls (Figure 4), indicating more rapid gastric emptying in this group. Paracetamol AUC concentration was negatively correlated with GIGD (rho −0.326, *p* = .008), but positively correlated with fasting glucose (rho 0.279, *p* = .024), 2‐h glucose (0.411, *p* < .001) and HbA1c (rho 0.427, *p* < .001). It did not correlate with the incretin effect (rho −0.140, *p* = .274) or rectal pressure for first sensation (rho 0.216, *p* = .1).

### 3.3 Clinical correlations

Rectal sensitivity for the earliest sensation was not correlated with the incretin effect (rho = −0.204, *p* = .106), but with GIGD (rho −0.341, *p* = .005). There was no difference in the incretin effect for those with rectal pressure for the earliest sensation <3.0 kPa (36 ± 24%) and for those with rectal pressure for first sensation ≥3 kPa (29 ± 25%), *p* = .286. The respective values for GIGD differed between 47 ± 25% (<3.0 kPa) and 34 ± 24% (≥3.0 kPa), *p* = .051. No significant associations were found between the incretin effect or GIGD and other neuropathy tests, including evoked potentials.

**FIGURE 5:**
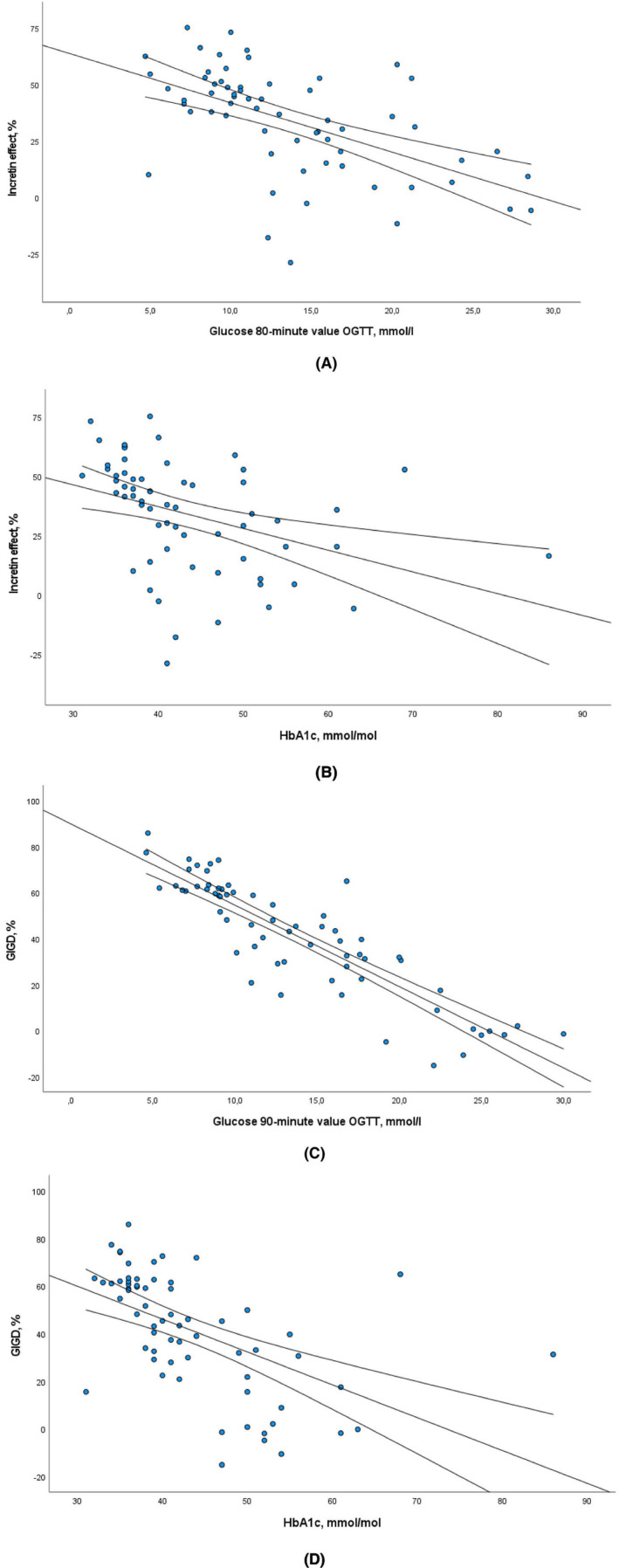
Correlations for the incretin effect and GIGD with glucose values and HbA1c. (A) Correlation for the incretin effect with OGTT glucose value at 80 min, rho −0.616, *p* < .001. (B) Correlation for the incretin effect with HbA1c, rho −0.573, *p* < .001. (C) Correlation for GIGD with OGTT glucose value at 90 min, rho −0.873, *p* < .001. (D) Correlation for GIGD with HbA1c, rho‐0.653, *p* < .001.

Both the incretin effect and GIGD were significantly correlated with all OGTT glucose values, with the strongest correlation with the 80‐min value of the incretin effect (rho −0.616, *p* < .001) and the 90‐min value of GIGD (rho −0.873, *p* < .001). Both the incretin effect and GIGD were also correlated with HbA1c (rho −0.573 and rho −0.653 respectively, both *p* < .001), see Figure 5A–D.

## 4 DISCUSSION

Rectal hyposensitivity, uncovered in both longstanding and early diabetes, was not associated with the incretin effect but was associated with GIGD. Additionally, an association was confirmed between both the incretin effect and GIGD, and dysglycaemia and duration of diabetes. The findings indicate a possible role for autonomic nerve dysfunction in the impact of gastrointestinal factors on glucose disposal other than the incretin effect.

### 4.1 Gut hyposensitivity in early stages of diabetes

We have previously reported and discussed the findings of rectal hyposensitivity in early stages of diabetes, indicating early onset of visceral hyposensitivity in the gut wall, supporting the presence of distorted neuronal transmission.^17^

### 4.2 No association with the incretin effect, but with gastrointestinal‐mediated glucose disposal

We did not find any association between visceral sensation and the incretin effect. This is indeed interesting as it could indicate that intact neuronal transmission is not a prerequisite for the incretin effect. However, reduced rectal sensation was associated with GIGD, which may be a highly relevant, but incompletely mapped, physiological parameter. GIGD is to an extent dependent on incretin hormones for insulin release, but also on factors such as glucagon secretion, first‐pass hepatic glucose uptake from the portal vein, tissue glucose uptake, hepatic insulin uptake, gastric emptying and gut motility, and activity on the gut–brain or liver–brain axis, including local afferent nerves.^6^, ^7^, ^20^, ^21^ Decoding which of the remaining factors mediated the correlation with visceral hyposensitivity is a potentially important area of future research and yields the possibility of discovering new relevant factors, that may serve as future therapeutical targets.

One possible explanation for the discrepancy between associations for rectal hyposensitivity with GIGD and the incretin effect could be that despite the limited amount of GLP‐1 reaching circulation, it could be sufficient to elicit a proper incretin effect. This is supported by evidence of a preserved incretin effect after complete surgical denervation of the pancreas following pancreas transplantation.^22^ Interpolation of these results suggests that despite an adequate incretin effect, GIGD was reduced in the transplanted group.^6^

Diminished GIGD, but preserved incretin effect has earlier been reported in truncal vagotomised subjects, with higher early GIP response, higher peak intact GLP‐1 levels and faster gastric emptying in those who were vagotomised.^6^ The fact that both GIP and GLP‐1 are found to increase in vagotomised subjects opens a hypothesis of increased hormone secretion, compensating for a reduced effect through neural transmission.

In the vagotomised subjects, a lack of glucagon suppression was reported following OGTT, similar to early steps in the development of type 2 diabetes.^23^ This is supported by a study where lean people with well‐controlled type 2 diabetes were found to have normal incretin effect but compromised GIGD, associated with hyperglucagonaemia after OGTT.^24^ Moreover, a study in pancreatectomised men has revealed that postprandial hyperglucagonaemia may be a gut‐dependent phenomenon.^25^

A possible association with GIGD, but not the incretin effect, could also point to a more important role for neuronal transmission in the central mechanisms of GLP‐1. This is supported by the abovementioned cohort of vagotomised subjects, where exogenous GLP‐1 did not have an effect on ad libitum eating or gastric emptying compared to controls, but showed the same reduction in postprandial glucose.^26^

Another proposed mechanism for the reduction of GIGD related to parasympathetic withdrawal was shown in people with cirrhosis, characterised by decreased hepatic first‐pass glucose uptake.^27^ In support of this, a review has suggested the presence of abnormal sympathetic dominance in prediabetes.^12^

Other possible explanations for a lesser role of intact vagal transmission in the incretin effect include the fact that GIP does not exhibit the same dependence on neuronal transmission as GLP‐1 and that GIP may be more central to the incretin effect than initially postulated.^7^, ^9^

To our knowledge, only one study has previously investigated whether a diminished incretin effect could be a consequence of diabetic autonomic neuropathy, reporting possible participation of autonomic neuropathy in the incretin effect through modulation of GIP secretion and hepatic insulin extraction.^28^

We noted with interest that both groups of diabetes had more rapid increase and higher paracetamol AUC concentration at 70 min, indicating more rapid gastric emptying than the control group. Physiological hyperglycaemia is shown to slow gastric emptying in people without diabetes and in insulin dependent diabetes.^29^ Our results are supported by studies of similar cohorts in terms of diabetes duration and without severe complications, reporting accelerated gastric emptying, regardless of glycaemic status.^30^, ^31^ Several reasons for accelerated gastric emptying have been proposed including hyperglycaemia‐induced oxidative stress leading to neural abnormalities, hyperplasia of interstitial cells of Cajal and smooth muscle hypercontractility, and loss of function of hormones responsible for slowing gastric emptying.^32^ The latter is proposed to be explained in part by a lack of small intestinal sensitivity to nutrients.^33^ It has previously been reported that a distal intestinal glucose exposure, compared to a proximal, was associated with an increased incretin effect and GIGD, in the same way as after surgical gastric bypass.^34^ In contrast to this, we report a negative correlation between gastric emptying and GIGD. This may strengthen the notion of a bidirectional relationship between gastric emptying and glycaemia, and could indicate that other factors that are associated with reduced GIGD contribute directly or indirectly to the gastric emptying rate.

### 4.3 Association with the degree of dysglycaemia and duration of diabetes

We substantiate earlier findings of an association between the degree of dysglycaemia, duration of diabetes and the incretin effect. The estimation of the incretin effect varies with the amount of glucose administered, gastric emptying rate, and whether the estimation is based on insulin, c‐peptide or insulin secretion rate (ISR). Estimates based on ISR or insulin tend to be lower than those based on c‐peptide, mainly due to hepatic insulin extraction. Compared to similar studies, estimating the incretin effect using c‐peptide after 75 g of glucose ingestion, the incretin effect was reported at 59%–64% in healthy adults, 6% in type 2 diabetes and 37% in a group without diabetes but with BMI of 29.^9^ Our rates of GIGD are also comparable to previous results, reported to be around 60% in healthy subjects, and in the range of 10%–30% in people with type 2 diabetes.^21^ The comparative similarity with our study strengthens the confidence in our results.

Our findings are novel in showing a continuum in the incretin effect, from healthy subjects to people with longstanding diabetes. A previous study, including people with prediabetes, calculating the ISR has reported an incretin effect of 20% in type 2 diabetes, 45% in prediabetes and 59% in matched controls, but in a cohort with a mean age of 50 years and a BMI of 30.^35^ Hence, our results underscore the gradual progress of a reduced incretin effect, in synchronicity with the level of dysglycaemia, starting from well within normal HbA1c levels, towards frank diabetes.

### 4.4 Strengths and weaknesses

The composition of our study population may explain the low prevalence of neuropathy and few between‐group differences. Exclusion criteria, especially use of GLP‐1 analogues and insulin, and proliferative retinopathy, may have led to selection of participants with longstanding diabetes with lean phenotype, an acceptable HbA1c and overall few microvascular complications. Also, in line with the results from the OGTT, some healthy volunteers had to be re‐classified as early diabetes, and some controls satisfied the criteria for prediabetes, making our groups more homogenous than we would have anticipated. Consequently, the external validity was impaired as our groups were less representative of the general diabetes population. On the contrary, this highlights the gradual onset of hyperglycaemia in this age group, and the results contribute to new knowledge of the incretin effect in an age group that exhibits an increasing prevalence of type 2 diabetes worldwide, mainly due to a longer life expectancy. Finally, with regard to the impact of low BMI on external validity, this may also be seen as a strength, more certainly attributing the reduced incretin effect to diabetes, as obesity itself has also been shown to deteriorate the incretin effect.^23^

Glucose was not measured immediately before or during rectal balloon distention or other neuropathy tests, and no examinations were done while performing a euglycemic clamp. Evidence concerning the effect of hyperglycaemia on rectal sensitivity is conflicting.^36^, ^37^ We cannot exclude glucose levels may have affected our results on the day of examinations.

Investigating afferent nerves of the rectum can only be a surrogate for nerves relaying information on gut hormones. We also acknowledge that plasma levels of GLP‐1 secretion in response to different nutrients have shown a substantial inter‐individual variation.^38^ Still, there are similarities in both structural and physiological attributes between mucosal afferents in the rectum and vagal afferents found more proximally, providing a rationale for the rectal balloon distention test.^39^, ^40^

The paracetamol absorption test is a feasible test evaluating gastric emptying, but still suboptimal, with several limitations as a surrogate marker.^41^ Hence, results on differences between groups on gastric emptying must be considered with caution.

Finally, our power estimates were uncertain due to the unknown effect size for the rapid rectal balloon distention test. With many non‐significant tendencies in this study, it is possible that a larger sample size would have avoided type II errors.

## 5 CONCLUSIONS

No associations were detected between CAN, sudomotor function or rectal sensitivity with the diminished incretin effect. However, rectal sensitivity, independent of diabetes duration, was associated with GIGD, alluding to a potential link between autonomic nerve function and gut‐derived mechanisms governing glucose homeostasis. Finally, we confirm that the incretin effect deteriorates with a longer duration of diabetes and worsening of glycaemic control, in a cohort of older individuals. The novel results warrant further studies on the potential role of the autonomic nervous system in all aspects involved in gastrointestinal‐mediated glucose disposal.

## AUTHOR CONTRIBUTIONS

**Sondre Meling:** Conceptualization (equal); data curation (equal); formal analysis (lead); funding acquisition (equal); investigation (lead); project administration (lead); resources (equal); writing – original draft (lead); writing – review and editing (equal). **Erling Tjora:** Conceptualization (lead); investigation (equal); methodology (equal); project administration (equal); supervision (equal); writing – review and editing (equal). **Heike Eichele:** Data curation (equal); investigation (equal); supervision (equal); writing – review and editing (equal). **Rasmus B. Nedergaard:** Data curation (equal); formal analysis (equal); investigation (equal); software (equal); writing – review and editing (equal). **Filip K. Knop:** Conceptualization (equal); investigation (supporting); methodology (supporting); resources (supporting); supervision (equal); writing – review and editing (equal). **Niels Ejskjaer:** Conceptualization (equal); funding acquisition (equal); methodology (equal); supervision (equal); writing – review and editing (equal). **Siri Carlsen:** Conceptualization (supporting); methodology (supporting); supervision (equal); writing – review and editing (equal). **Pål R. Njølstad:** Funding acquisition (equal); methodology (supporting); resources (equal); supervision (equal); writing – review and editing (equal). **Christina Brock:** Conceptualization (equal); methodology (equal); software (equal); supervision (equal); writing – review and editing (equal). **Eirik Søfteland:** Conceptualization (lead); funding acquisition (equal); investigation (equal); methodology (equal); project administration (lead); resources (lead); software (lead); supervision (lead); writing – review and editing (lead).

## CONFLICT OF INTEREST STATEMENT

No authors declare a conflict of financial or competing interest and the activity or relationship that might bias or be perceived to bias, their work.

## ETHICS STATEMENT

The study was approved by the Western Norway Regional Ethics Committee for Medical and Health Research Ethics (REK Vest 2018/#1790). All procedures were carried out following the Declarations of Helsinki and its amendments, and all participants signed an informed consent following oral and written information.

## Data Availability

The data supporting this study's findings are not openly available due to national regulations, but are available from the corresponding author upon reasonable request.
